# Shape and fractures of *carina sterni* in chicken genotypes with different egg deposition rates reared indoor or free-range

**DOI:** 10.1038/s41598-023-49909-1

**Published:** 2023-12-15

**Authors:** Domitilla Pulcini, Simona Mattioli, Elisa Angelucci, Wei Chenggang, Alice Cartoni Mancinelli, Riccardo Napolitano, Federico Sirri, Raffaela Piscitelli, Cecilia Mugnai, Cesare Castellini

**Affiliations:** 1 Council for Agricultural Research and Economics, Animal Production and Aquaculture, 00015 Monterotondo, Rome, Italy; 2 Department of Agricultural, Food and Environmental Sciences, University of Perugia, 06100 Perugia, Italy; 3 Department of Agricultural and Food Sciences, Alma Mater Studiorum - University of Bologna, 40064 Ozzano dell’Emilia, Italy; 4 Department of Veterinary Sciences, University of Turin, 10095 Turin, Italy

**Keywords:** Physiology, Zoology, Animal behaviour, Animal physiology

## Abstract

Commercial laying hens have high frequency of damage to the keel bone (KB), which causes negative effects on health and welfare. KB damage may consist in fractures (KBF) and deviations (KBD). The aim of the present study was to compare the KB shape, by means of Geometric Morphometric, and the occurrence of fractures in different chicken genotypes reared either free-range (FR) or in enriched cages. Moreover, the relationship between KB shape, genotype and rearing system was analysed. Sixty birds/genotype (2 Italian local breeds, *Bionda Piemontese* and *Robusta Maculata,* their crossbreeds with Sasso and Lohmann Brown) were used. All the hens fed the same commercial feed throughout the trial. Body weight, egg production, feed intake and mortality were recorded from 25 to 66 weeks of age. Ca intake (IN) and output (OUT) were estimated and Ca OUT/IN was calculated. FR affected the occurrence of KB deviations but not the shape, whereas the fractures were mainly affected by genotype. Local breeds had a lower prevalence of KBF with similar level of KBD but with different shapes. Crossbreeds seemed to be a suitable compromise between egg deposition rate and occurrence of KB damages.

## Introduction

The European Commission (EC), in the Farm to Fork Strategy, undertook a comprehensive evaluation of the animal welfare legislation^1^, including Council Directive 1999/74/EC^2^, which lays down minimum standards for the protection of laying hens. In addition, a European Citizen Initiative (ECI) ‘end the cage age’^3^, calls for banning the use of cages in laying hens and other livestock species (i.e. pigs, calves, rabbits)^4^. In the light of such changes, EC requested EFSA to describe the current husbandry systems and practices of keeping laying hens, pullets and layer breeders, related welfare consequences, animal-based measures (ABM), and hazards, with the aim of preventing or correcting them^5^.

The high frequency and severity of damage to the keel bone in commercial laying hens represents one of the greatest welfare issues, as suggested by the EFSA report^4,5^, which considered them as an important ABM, causing negative states such as pain, health, welfare, reduction of production performance and the egg quality of laying hens. The different susceptibility of commercial genetic strains to KBD is shown by different authors^6–9^; however, the KB status in local breeds is largely unknown and is limited to some egg-type breeds^10^. A recent study, comparing the Red Jungle fowl hens with Leghorn hens showed huge differences in prevalence of KBF^8^.

Keel bone (KB) damage is a general term that can be subdivided into 1) fractures (KB fractures [KBF]), intended as breaks in the bone that typically manifest as a callus around the fracture site, and 2) KB deviations [KBD], characterized by sharp, unnatural bending from a two-dimensional plane^11^. These abnormalities, mainly KBD, are often subjectively evaluated^4^ and the different severity of deviations makes comparison difficult.

The number of affected birds within commercial flocks is affected by many factors^12^ and can range between 80 and 96%.

Selection for early sexual maturity and high egg production in commercial lines has led to an increase in bone fragility and susceptibility to fractures due to the high calcium requirement for the formation of eggshells^13^. Accordingly, production performance, genetics, nutrition and rearing system are strictly linked and affect KB damages. The usual explanation for the cause of fractures is the high calcium demands of hens required for egg production (approximately 320 eggs/year, i.e. nearly an egg per day), such requirement induce reabsorption or the breakdown of the bone matrix and the release of calcium. The entire process is believed to leave bones weak and brittle^14–16^. Although not a cause of fractures by itself, the resulting poor bone health leaves the hen relatively susceptible to KBF from direct causes such as collisions with housing objects, which in turn may depend on the housing system (i.e. perches^17^, free-range) and animal stocking density^18^.

Theoretically, the effect of Free Range (FR) on KB should be positive for the combined effect of exercise and natural light on vitamin D activation and the resulting improvement of Ca metabolism^15^. It is reported that exercise can improve KB consistency by enhancing bone calcium deposition^19^. However, the effect of free-range is not widely studied and conflicting results are present because many factors are confounding (genotype, grass intake, season, uncontrolled environment, exposition to sunlight).

In this context, the aim of the present study is to:

- Compare the keel bone shape in chicken genotypes characterized by different egg deposition rates (commercial, local breed and their crossbreed) by means of an objective tool, Geometric Morphometric techniques, allowing unprejudiced comparisons among different groups^20^.
- Describe the relationship between shape alterations of keel bone and variables potentially affecting it, such as rearing system, genotype, egg deposition rates, and calcium balance.
- Propose an objective evaluation of keel bone deformations in laying hens, instead of the use of subjective evaluations, which also require a trained staff to obtain reliable results.

## Results

### General results

Hen weights were significantly different among genotypes (Tables S1 and 1; Welch F test between groups, F = 51.5, P < 0.001), pairwise comparisons are reported in Table 1.

**Table 1:** Percentage and number of fractures (in brackets) and deviations (n = 15/genotype/rearing system), hen weight (mean ± S.D., n = 30/genotype/rearing system), centroid size (mean ± S.D., n = 15/genotype/rearing system), and egg deposition rates (n = 30/genotype/rearing system) at the 35th week of different hen genotypes reared in enriched cages and free-range system.

Genotype	Rearing system	Fractures, % on total fractures, (n)	Deviations (%)	Hen weight (kg)^1^		CS (cm)		Egg deposition rate (%)^2^
Lohmann Brown	EC	21.0 (13)^bc^	8.0^a^	1.88 ± 0.14^a^	1.91 ± 0.17	15.43 ± 1.92^a^	15.14 ± 0.53	91.1 + 9.15^f^
	FR	41.9 (26)^c^	40.0^b^		1.86 ± 0.13		15.60 ± 2.43	94.8 + 9.22^f^
*Robusta maculata*	EC	1.6 (1)^a^	8.0^a^	2.75 ± 0.42^b^	2.65 ± 0.44	17.87 ± 0.90^b^	18.29 ± 0.74	57.0 + 6.10^c^
	FR	1.6 (1)^a^	12.0^b^		2.88 ± 0.39		17.37 ± 0.88	50.9 + 5.5^b^
*Bionda piemontese*	EC	8.1 (5)^a^	0.0^a^	1.98 ± 0.23^a^	2.03 ± 0.24	15.21 ± 1.30^a^	15.17 ± 1.62	53.9 + 4.9^cd^
	FR	1.6 (1)^a^	8.0^b^		1.93 ± 0.23		15.26 ± 0.96	42.1 + 4.6^a^
Crossbred* Robusta maculata*	EC	1.6 (1)^a^	0.0^a^	3.15 ± 0.28^c^	3.39 ± 0.78	18.64 ± 1.29^b^	18.09 ± 1.58	73.2 + 6.3^e^
	FR	0.0 (0)^a^	8.0^b^		3.07 ± 0.29		18.82 ± 1.29	69.7 + 6.2^cd^
Crossbred *Bionda piemontese*	EC	14.0 (9)^ab^	8.0^ab^	2.74 ± 0.29^b^	2.77 ± 0.21	16.10 ± 1.11^a^	16.14 ± 0.79	71.4 + 6.5^de^
	FR	8.1 (5)^a^	8.0^ab^		2.70 ± 0.39		16.05 ± 1.52	63.2 + 6.4^cd^

*EC* enriched cage, *FR* free-range, *CS* centroid size.

^a–f^On the same column P < 0.05.

^1^Hen weight at 66 weeks of age. ^2^Egg deposition rates at 35th week.

The number of KBF was affected by genotype (X^2^ = 3.78; P = 0.043), whereas the number of KBD was affected by the rearing system (X^2^ = 5.45; p = 0.020) with hens reared in FR showing the highest value.

Centroid size (CS) was different among genotypes (ANOVA, df = 4, F = 11.4, P < 0.01), and pairwise comparisons are reported in Table 1. Hierarchical Procrustes ANOVA produced coherent results, as the main effect of genotypes was statistically significant (F = 9.64, P < 0 0.05), while differences in bone size between lines reared in different systems were not significant (F = 0.21, P = 0.67), as well as the interaction between the two factors (F = 2.05, P = 0.11).

Concerning Hierarchical Procrustes ANOVA performed on shape (Procrustes coordinates), the main effect of genotype was significant (F = 1.56, P < 0 0.001), the effect of the rearing system was not significant (F = 1.13, P = 0.29), and the interaction between the two effects was significant (F = 1.27, P < 0.05). Hen productivity (“high”; egg deposition rate ≥ 90%, or “low”) was added as an extra-effect to the ANOVA (F = 1.67, P < 0.05).

Table 2 shows the results on Ca balance, estimated using the ratio between the Ca intake (IN, only feed) and the Ca retained in the eggshells (OUT). Data demonstrated that Ca ratio (OUT/IN), also corrected for the possible intake of soil and stones, was similar in FR and EC groups; however, a significantly higher Ca mobilisation was found in LB when reared in free-range, respect to other genotypes. This trend is partly due to lower feed consumption of hens in FR system (from 0 to about 12%) which led to a greater demand for Ca, taken from the bones.

**Table 2:** Ca intake (IN, g d^–1^), Ca in the eggshell (OUT, g d^–1^) and Ca OUT/IN of different hen genotypes (n = 3/genotype/rearing system) reared in enriched cages and free-range system.

Genotype	LB		RM		BP		RMxS		BPxS		RMSE
Rearing system	EC	FR	EC	FR	EC	FR	EC	FR	EC	FR	
Intake Ca (intake)	6.10^c^	5.37^b^	5.02^b^	5.02^b^	4.91^ab^	4.31^a^	6.02^c^	5.73^bc^	5.52^bc^	5.60^bc^	0.52
Output Ca (output)	5.72^e^	5.90^e^	2.94^b^	2.68^b^	2.75^ab^	2.00^a^	4.05^d^	3.80^cd^	3.88^cd^	3.56^c^	0.38
Ca output/intake	0.92^d^	1.10^d^	0.59^b^	0.54^ab^	0.57^ab^	0.47^a^	0.67^bc^	0.67^bc^	0.71^c^	0.64^bc^	0.08
Out/In corrected	0.92^d^	1.00^d^	0.59^b^	0.49^ab^	0.56^ab^	0.43^a^	0.67^bc^	0.61^bc^	0.70^c^	0.57^bc^	0.09

*LB* Lohmann Brown, *RM* Robusta Maculate, *BP* Bionda Piemontese, *RMxS* Robusta mMculata x Sassò, *BPxS* Bionda Piemontese x Sassò, *RMSE* root mean standard error, *EC* enriched cages, *FR* free-range.

^a–e^On the same row P < 0.05.

### KB shape

**Figure 1:**
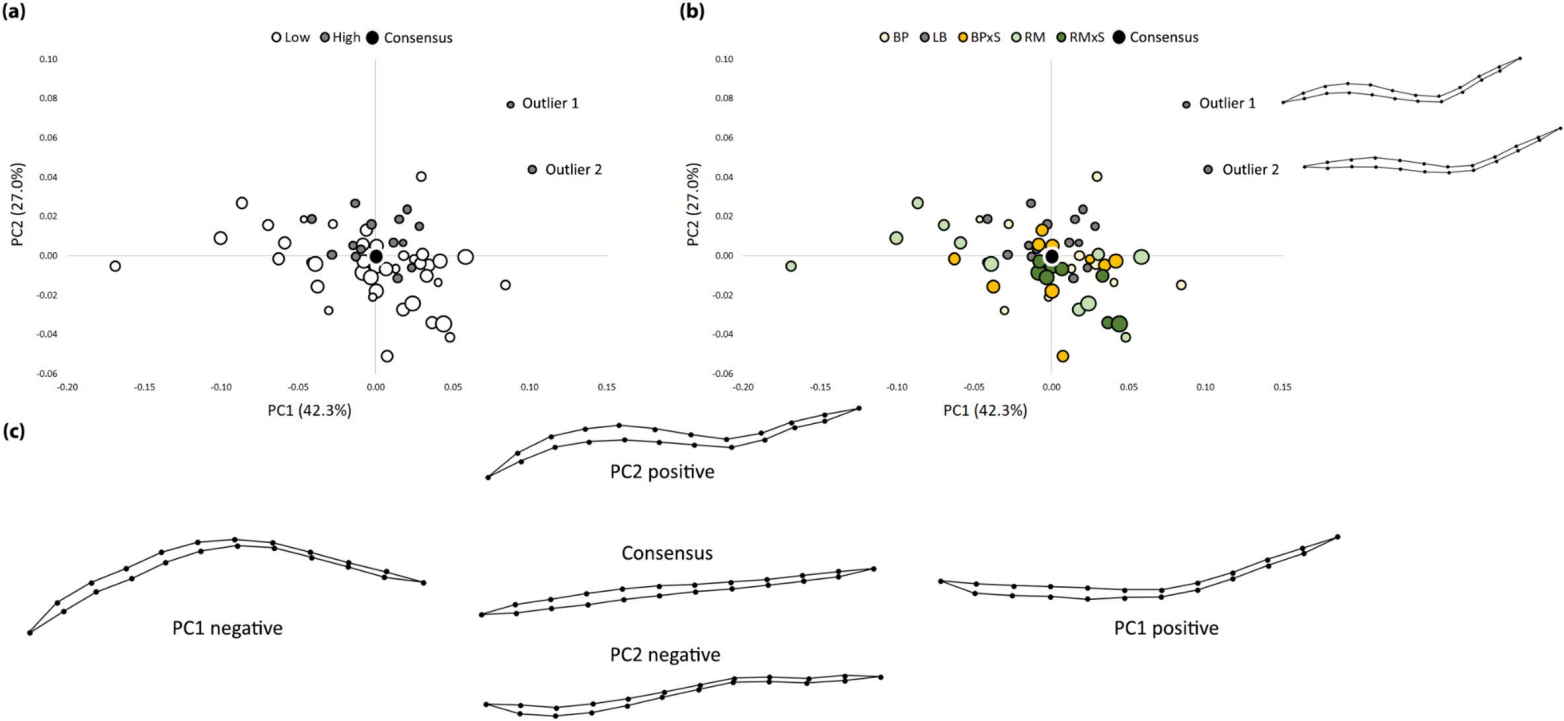
Principal component analysis plot. Grouping variables: (**a**) productivity (low and high); (**b**) genotypes (bubble size according to live weight, outlier shape shown in each spline); (**c**) shape variation along PC1 and PC2. Consensus: a set of landmarks intended to represent the central tendency of an observed sample, computed to minimize the sum of squared Procrustes distances from the consensus landmarks to those of the sample.

Hens were distinguished in the PCA plot according to productivity (high or low) and genotype (Fig. 1). The first two PCA axes accounted for 69.3% of the total shape variation.

Low and high productive lines were highly overlapped in the morpho-space, but the individuals belonging to the high one (LB) were concentrated around the centre of the plot (Fig. 1a), with two outliers at the positive PC1 (42.3%) extreme, characterized by a deeply “S” deviated shape of the keel bone (Fig. 1b). Low productive genotypes were highly scattered, with very different shape configurations: along PC1, the “C” curvature of the keel bone varied, while along PC2 (27.0%) the “S” curvature showed deep variation. Different genotypes were highly overlapped in the morpho-space, and a clean pattern was not identified (Fig. 1b). In general, crossbred (BPxS and RMxS) tended to occupy the central region of the plot, nearest to the consensus configuration.

The correlation between hen final weight and shape (individual scores along the first PCA axis, PC1) was very weak (r = − 0.03) and not significant (p = 0.81), as well as the correlation between size of the keel-bone (centroid size, CS) and shape (PC1 scores) (r = − 0.03, p = 0.82). Therefore, differences in hen weight and keel-bone size can be excluded as factors determining the observed shape variation among groups.

### Relationship between keel bone shape and other variables

**Figure 2:**
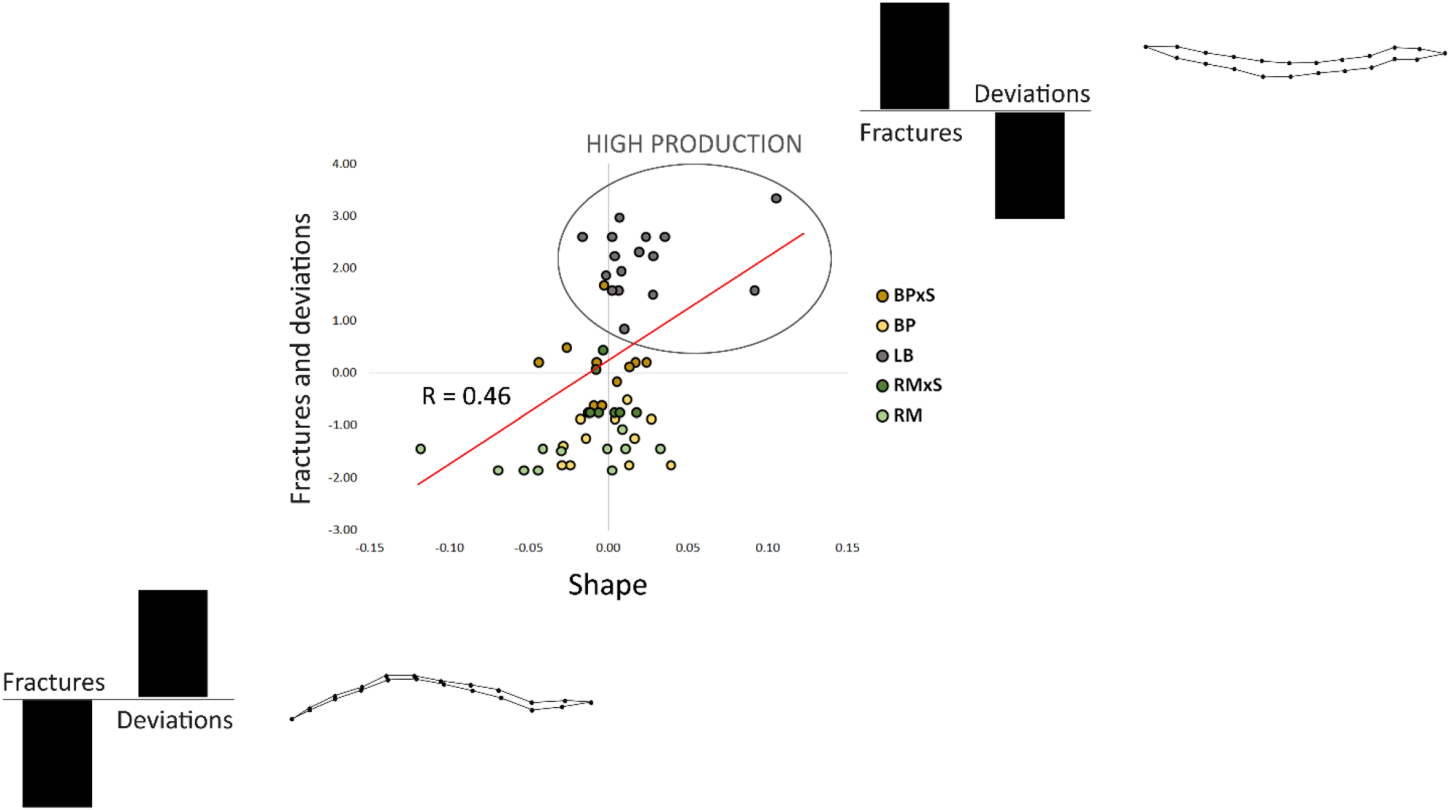
Plots of coupled partial least square latent vectors of shape and deformations of *carina sterni* showing the relationship between them. Highly productive hens (LB) are inside the grey circle. The splines depict shape variation corresponding to different patterns of combinations of the variable “fractures” and “deviations”.

A significant covariance between the keel bone shape and the degree of bone deformation (measured as the number of fractures and deformations observed for each individual) was detected with PLS (R = 0.46; p < 0.05) (Fig. 2). The low “R” value was probably due to the recording system of deviations, as they were recorded just as presence/absence (0/1). The model yielded by the first two latent vectors represents the coupled changes in shape and deformation. The extremes of the linear pattern were characterized by the occurrence of opposite patterns of fractures and deviations: a minimum in fractures and a maximum in deviations was associated with a thin *carina sterni* of the keel bone, with a remarkable “S” shape. This pattern of shape deformations was typical of the RM genotype. At the opposite extreme, a maximum of fractures and a minimum of deviations was associated with a “C” shaped thicker keel bone. Specimens belonging to the LB (highly productive) genotype were positioned at this extreme of the linear pattern. It should be noted that the thicker shape of KB in hens (upper right) with more fractures and less deviation is partly due to the formation of bone callus.

**Figure 3:**
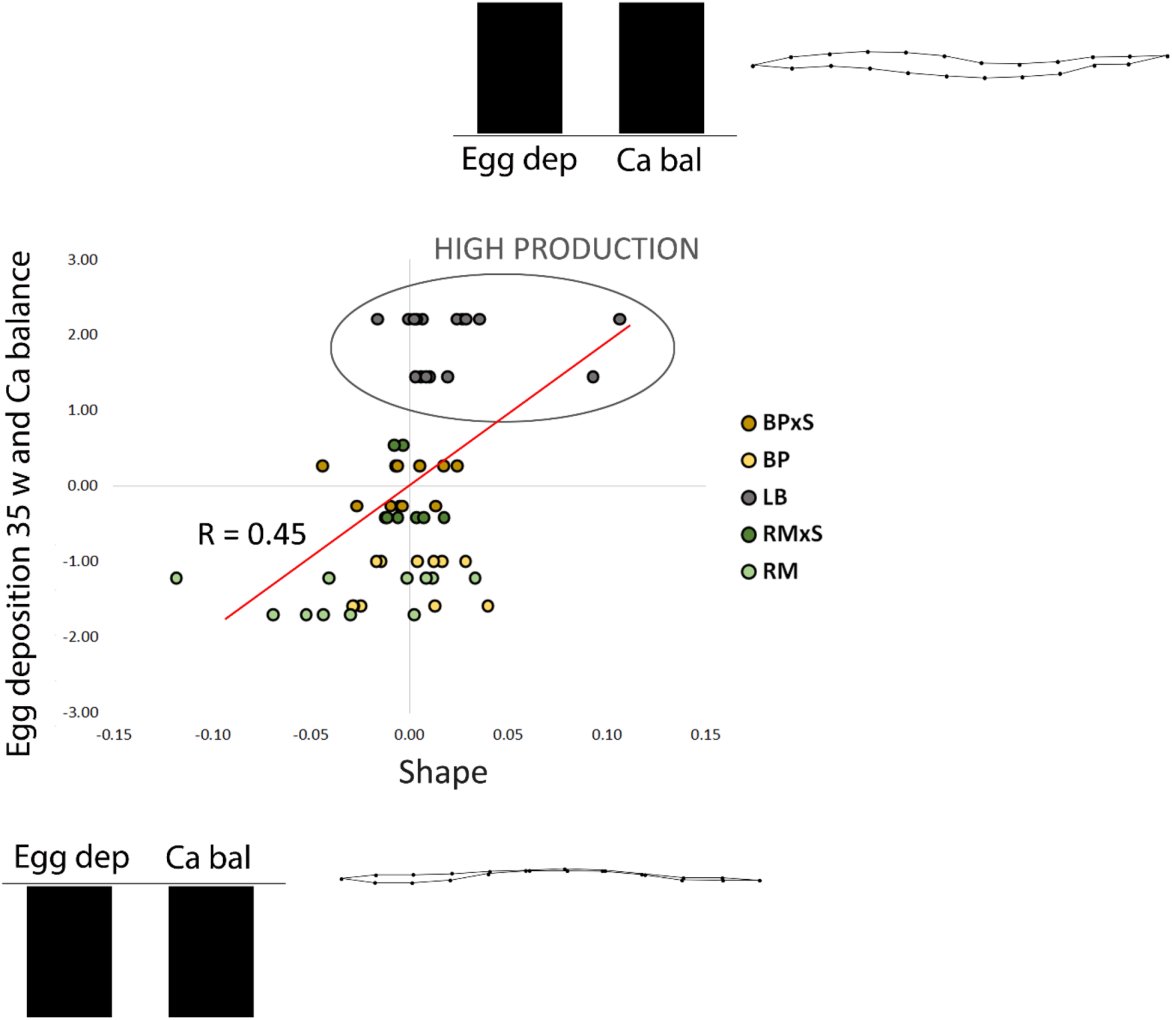
Plots of coupled partial least square latent vectors of shape of the keel bone: egg deposition rates at the 35th week (Egg dep), and Ca imbalance (Ca bal), showing the relationship between them. Commercial hybrid hens (LB) are positioned inside the grey circle. The splines depict shape variation corresponding to the pattern of variables in the histogram (at the extremes of the plot).

When shape was correlated with egg deposition rates at the 35th week and Ca balance, a significant (R = 0.46, p < 0.05) relation was found (Fig. 3). A highly productive genotype (LB) was isolated at the positive extreme of the linear pattern, characterized by the highest values of egg deposition rates and Ca balance. The keel bone was thicker, and the “C” curvature was concave. At the negative extreme, the RM genotype was characterized by a thinner keel bone, with a convex “C” shape, and by lower values of egg deposition rates and Ca balance.

## Discussion

To the best of our knowledge, this is the first study that objectively compare the KBF, KBD and keel bone morphology of a commercial hybrid (LB) with local breeds (BP and RM) and their crossbreds (BPxS and RMxS). In these different genotypes, the egg deposition rate, the rearing system and the balance of Ca (output/input) have also been assessed.

It should be noted that the maintenance of local breeds is relevant for supporting biodiversity, but these chickens have been progressively abandoned due to the low productivity with respect to commercial hybrids^21–24^. A common strategy to increase the performance of these breeds is the cross-breeding with more productive chicken lines^25,26^. This reproductive procedure gives cockerels with higher growing rate^22^ and hens with higher egg deposition rate and egg weight^27,28^.

Geometric Morphometric were also used to study *carina sterni* by Gündemir et al.^29^ but only for comparing the KB of different birds species.

### Relationship between KB abnormalities

Our results support and better describe the role of the rearing system and genotype as main factors in shaping the keel bone and causing different types of damage. The relationship between these KB abnormalities turned out to be complex, in particular the usual keel bone shape of laying hen essentially follows a straight line; however, our results showed that some deviations may occur (Fig. 1a,b). These deviations from a straight configuration could also be physiological in heritage poultry breeds with low productive performance (Fig. 2). Kittelsen et al.^8,30^ found the presence of some deviations also in hens of jungle fowl, the ancestor of modern chicken lines. The same authors confirm that the prevalence of fracture is different in the Red Jungle hens (10%) and in modern White Leghorns (69%).

The origin of KBD in hens is different from fractures because it is considered to be caused by the development of breast muscle (e.g. laying hens vs. broilers) and by exogenous factors causing a long-term pressure on the keel bone^31,32^.

Bokkers and Koene^33^ in meat-type chickens, found KBD at 12 weeks of age in 19.1% of a slow-growing genotype and in 2.4% of a fast-growing one suggesting that the structure of bone, and the muscoskeletal configuration (e.g. width, height and strength of *pectoral major*) could have a significant effect on KBD.

Stratmann et al.^34^concluded that the development of deviations does not occur during an acute event, but takes place over a period of time due to bone remodelling and interaction with the muscoskeletal system Some authors^35^ reported that during perching the force acting on KB to support the body weight is very high (five times higher than on the footpad, about 6 N/cm^2^). Other reports^36^ suggest that the period during which injuries occur is very early and before the peak of egg deposition.

We did not measure the KBD during early stages but the fact that free-range modified the prevalence of KBD suggests that some modifications occurred late (after 18 wk.) and that the intrinsic structure of the KB and the presence of outdoor runs had a relevant effect. Clearly, wing flapping and other kinetic activities, which are higher in hens of the FR group, could be a further reason for the differences found in KBD^37^.

### Correlation between fractures and deviations

As far as we know, the relation found between KBD and KBF and the complex correlation between deviations and fractures were previously unrecognized, at least in the genotypes hereby analysed. Highly productive birds showed high fracture rates and lower deviation (with a C shape – upper right side of Fig. 2) and on the other hands less productive birds had the most notable deviations (S shape) and fewer fractures (lower left side of Fig. 2). It should be also noted that the greater thickness of *carina sterni* (upper right side of Fig. 2) could be affected by the progressive formation of bone callus following the KBF.

This relation is not obvious and deserves further in-depth analysis. It is possible that, once the deviation (mainly S shape) has taken place, the interaction between breast muscles and keel bone reduces the sensibility to KBF. At this regard, it is reported^38^ that highly productive hens also have a lower kinetic activity thus partially explaining the lower KBD.

Indirectly, Candelotto et al.^39^ confirmed this hypothesis since higher bone strength seemed to lower fracture susceptibility but had only a limited effect on keel flexibility and KBD. Anyway, the soundness of these results should be confirmed by other specific trials and verified also in other breeds/genotypes.

### Effect of rearing system

The rearing system (free-range-FR or enriched cages-EC) per se did not affect the keel bone shape or fracture occurrence, but only the number of animals with KBD. In free range system, two different and opposite factors interact: on the one hand, FR hens eat less feed (also less Ca – Table 2 and vitamin D_3_) due to the contemporary intake of raw materials (i.e. insects, heartworm, little stones) or just because they interact more with the environment than hens kept in enriched cage that eat also to overcome the stress induced by this farming condition. On the other hand, the effect of sunlight on the synthesis of vitamin D_3_ presumably increases the efficiency of Ca absorption^40^. Moreover, exercise may improve the consistence of the KB by enhancing bone Ca apposition^19^. In our experimental conditions, the reduction of feed intake probably predominated over the other factors, especially in highly productive hens.

Previous studies showed that FR hens, probably due to grass intake (about 100 g/hen/d), reduced their feed intake with respect to hens indoor reared, by about 25%^41^ and consequently lower amount of Ca, P and Vitamin D_3_^27,28^. However, in the present trial, there was any grass in the outdoor runs and thus the nutrients mostly derived from the supplied feed. Thus, in FR the reduced feed consumption (particularly evident in LB; about − 12%) did not depend on the grass intake but was probably linked to a more stimulating environment, which reduces the eating time^42^. Anyway, FR reduced the ingestion of key elements (Ca e P) needed for eggshell production as shown by the imbalance between Ca intake and output.

Other authors confirmed that FR could amplify the deficiency of calcium; Liu et al.^43^ found that pecking stones offered to FR hens induced a reduction of feed intake during egg deposition. Moreover, in different FR systems, a lower Ca content in the crop was found^44^. The hypothesis that the supplementary intake of soil and little stones could improve the Ca balance^45^ was also evaluated (Table 2, Fig. 3) but this potential additional contribution does not seem substantial^37^. These findings confirmed what Bestman and Wagenaar^46^ reported: that the amount of KBF in organic farms, which use highly productive genotypes, were comparable or worse than in conventional systems. In this circumstance (i.e. highly productive genotypes), Adhijkari et al.^27^ showed that additional dietary vitamin D had no effect on bone mineralization and eggshell strength.

Accordingly, Vitamin D_3_ and calcium metabolism in FR shall be better defined also if a first driver of this phenomenon seems to be the high egg deposition rate of hens. The relevance of sunlight exposure should be deeper analysed.

### Effect of hen genotype

The main findings on the impact of genotypes on keel bone damage, derived from this research, are the following: the commercial chicken hybrid (LB), although has a feed and Ca intake higher than local breeds, presented severe KBF, which most likely depends on the high egg deposition rate and on the difficulty of providing enough dietary Ca (OUT/IN Table 2; Fig. 3). Crossbreds showed lower prevalence of fractures than commercial hybrid and bone shapes similar to the consensus showing that the increase in deposition rate respect to local breeds seems be balanced by the feed intake.

A difference in KB abnormalities between breeds and commercial genotypes has been documented in previous studies^13,47^ and the present results are in line with these research, suggesting that the more selected hens also have worse bone health. Other papers underlined that several productive traits may be linked to KBF as for example early onset of laying. The LB genotype in this study started laying at about 19 weeks, while the other stains started to produce more than 4–5 weeks later; in this study we are not able to discriminate precocity from productive level because of collinearity between these variables. However, it is known that the ossification (bone formation) of the keel is not completed until 30–40 weeks of age^48^, possibly depending on the genotype, indicating that the modern hens start laying eggs many weeks before the keel bone is fully developed.

Our results confirm that all fractures were located in the caudal part of the keel (late in the ossification process), regardless of breed. Probably, all these productive factors (precocity, nutrition, egg deposition rate) interact each other.

The present results should be confirmed by further experiments with a higher number of keel bone samples and also involving other chicken genotypes. Longitudinal studies collecting the data before deposition and at different points during productive life of individual birds will permit a better assessment of the relationship between the multiple variables involved**.** Moreover, to explain the complex interactions of muscoskeletal configuration of the KB, 3D studies of KB morphometry will have many concerns for the future, to define clearly not only the KB damages but trying to explain the causes.

### General conclusion

In conclusion, the present study confirmed that the extent of KB damages in laying hens are complex and involves several productive factors (e.g. genetic, rearing system, feeding strategies).

In particular, highly productive hens showed the highest prevalence of KBF; furthermore, the egg deposition rate and the deficiency in Ca intake seemed to be one key factor in the KBF incidence.

Specifically, the use of a welfare-oriented rearing system (e.g. free-range), does not induce any improvement in bone health, at least in these strains. Indeed, free range determined a reduction of feed and Ca intake, likely, in such alternative rearing system a lower egg deposition rate may be considered a prerequisite for hens’ adaptation. However, also in less productive genotypes, FR increased the amount of KBD due to the higher activity (e.g. wing flapping etc.) of the bird in outdoor runs.

In the same time, FR probably requires feed with higher nutrient density (e.g. minerals as Ca, P) to overcome the reduction in voluntary feed consumption.

The local breeds (RM and BP), which showed about 60% of egg deposition, had a lower prevalence of KBF and similar KBD but with different shape with respect to LB. Crossbreedings (RMxS and BPxS), which are primarily used for increasing egg deposition of local breeds, seemed a suitable compromise between egg production and KB health. In addition, the present findings also highlighted that local breeds are not free from KB anomalies (i.e. they exhibited a different KB shape than consensus).

## Methods

### Experimental design, housing, and feeding

The trial was carried out at the experimental poultry facility of the Department of Agricultural and Food Science of the University of Bologna (Italy) from March 2021 to June 2022. All methods are reported in accordance with ARRIVE guidelines for the reporting of animal experiments. This study was conducted in accordance with the Guiding Principles in the Use of Animals and approved by the Animal Ethics Monitoring Committee of the University of Bologna (Protocol ID: 1102/2019).

**Figure 4:**
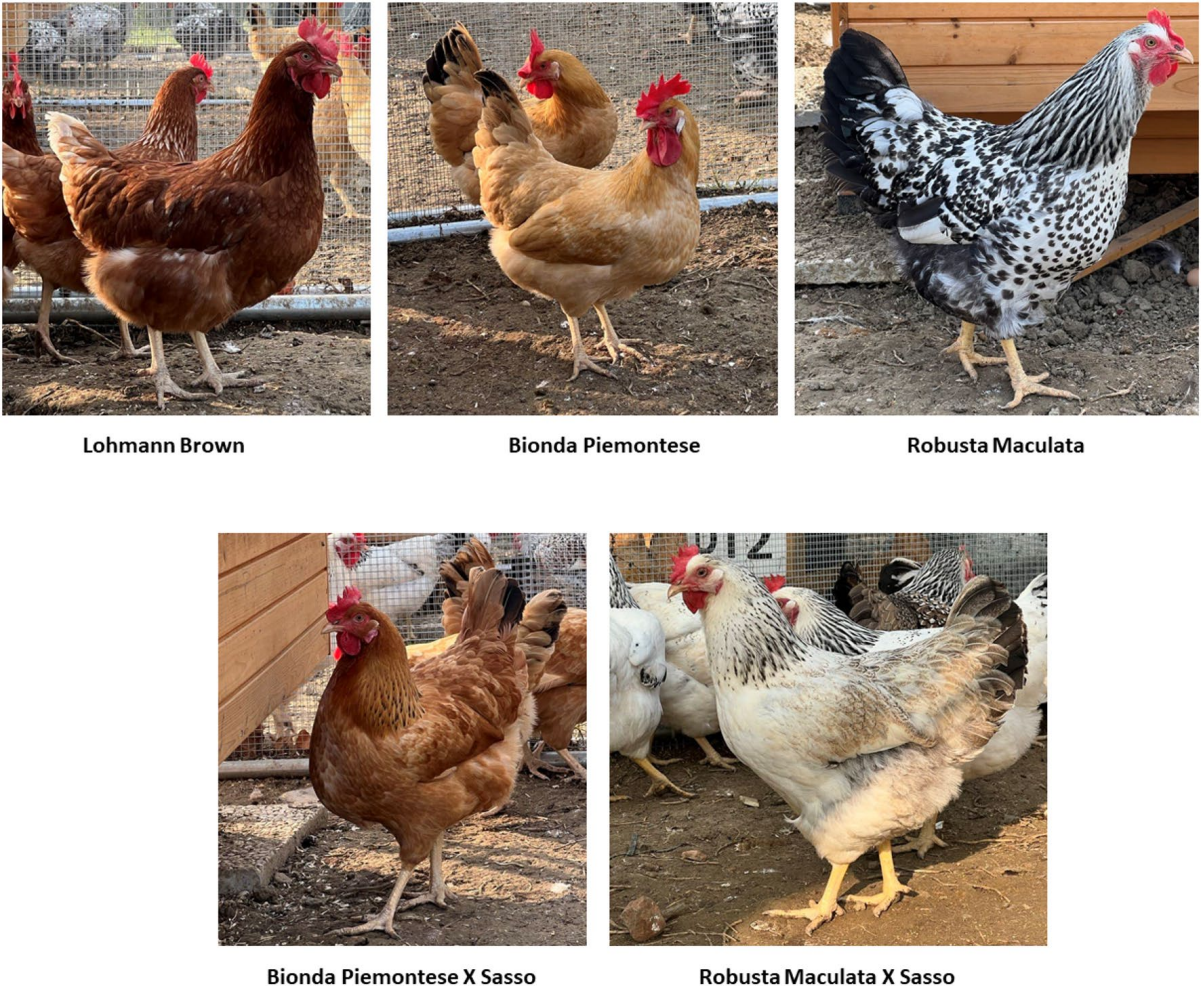
Pictures of genotypes used in the experimentation. Lohmann Brown (LB) as control, two local Italian chicken breeds, Bionda Piemontese (BP) and Robusta Maculata (RM), and their crossbreeds BP x Sasso (BP x S), and RM x Sasso (RM x S).

A total of three hundred female birds of the following genotypes (60 birds/genotype) were used (Fig. 4): two local Italian chicken breeds, Bionda Piemontese (BP) and Robusta Maculata (RM), their crossbreeds BP x Sasso (BP x S), and RM x Sasso (RM x S), and Lohmann Brown (LB). One-day-old female chicks of the five genotypes were vaccinated against Marek and Newcastle disease, and housed indoor on pen litter covered with wood shaving (about 3 kg/m^2^), from 1 to 18 weeks of age under the same environmental and managemental conditions. Temperatures and humidity were set up according to the guideline provided for the commercial hybrid for the brooding phase and throughout the pullet rearing duration (about 30 °C from 0 to 7 days of age and progressively reduced to achieve a 20–22 °C from 35 days onward; relative humidity ranged between 60 and 70%). All the pullets received the same commercial diet divided in 3 feeding phases and supplemented ad libitum in mash form. No environmental enrichment was provided in the pullet rearing and the photoperiod was set up in accordance with the guidelines of the commercial hybrid.

At 18 weeks of age, birds were randomly housed in two different rearing systems (Fig. S1): enriched cages and free-range system (3 replicates of 10 birds/genotype/rearing system). The experiment was arranged in a 5 × 2 full factorial model with 5 genotypes and 2 rearing systems. A total floor area of 1500 cm^2^ was provided per hen in each enriched cage. In the free-range system, the birds were allotted in fenced pens (1.6 m^2^/bird) including a poultry house (2000 cm^2^/bird) with nest. After the first week from the housing, the grass coverage of the pens was progressively reduced, and no grass was present after 10 days till the end of the trial. All the pens were fully exposed to sunlight and half of the pen surface was provided with shading nets. The photoperiod was maintained constant throughout the experiment duration at sixteen hours of light and eight hours dark in both systems. Indoor the hens received only artificial light, whereas outdoor the natural light was amended with the artificial one to achieve the same photoperiod duration in both systems. Perches were present in both the rearing systems and the space availability was about 30 cm/bird. Temperature and relative humidity were particularly affected by the season in outdoor system (T ranged between 35 °C during summer and about 0 °C in winter; R.H. ranged between 50 and 90%) and to a lesser extent in indoor system as no heating/cooling was used (T ranged between 30 °C during summer and about 12 °C in winter; R.H. ranged between 50 and 90%).

All the hens received the same commercial feed for the whole experimental period (Table 3). Feed and water were available on an ad libitum basis to all hens. Bird’s health status, mortality, and environmental parameters were daily checked during the whole experimental trial.

**Table 3:** Composition of the commercial diets according to different feeding phases.

	Pre-layer Wk 18 to 25	Layer 1 Wk 26 to 51	Layer 2 Wk 52 to 62	Layer 3 Wk 63 to 66
Ingredients, %				
Corn	34.53	31.03	46.12	51.23
Wheat	20.00	20.00	0.00	0.00
Soybean meal	3.00	10.47	8.93	7.01
Fullfat soybean	8.60	15.00	15.00	15.00
Sunflower meal	10.00	5.24	6.40	4.49
Corn gluten meal	5.00	5.00	5.00	5.00
Bran	12.32	0.00	4.00	4.00
Vegetable oil	1.00	1.00	2.13	1.13
Calcium carbonate	4.03	10.06	10.60	10.48
Dicalcium phosphate	0.13	0.59	0.28	0.11
Sodium bicarbonate	0.14	0.14	0.14	0.11
Sodium chloride	0.24	0.26	0.22	0.23
l-lysine	0.27	0.05	0.07	0.13
DL-methionine	0.05	0.06	0.06	0.06
MHA	0.05	0.23	0.20	0.20
Choline chloride	0.10	0.11	0.10	0.10
Phytase	0.06	0.08	0.08	0.07
Vitamin and mineral premix	0.34	0.34	0.34	0.34
Chestnut tannins	0.00	0.05	0.05	0.05
Natural yellow pigment	0.00	0.21	0.17	0.16
Emulsifier	0.05	0.05	0.05	0.05
NSP enzyme	0.05	0.00	0.00	0.00
Adsorb. micotox	0.05	0.05	0.05	0.05
Calculated nutrients				
Crude protein, %	16.00	17.85	17.00	15.85
Total fat, %	4.77	5.49	6.95	6.13
Crude fiber, %	5.01	3.60	4.00	3.60
Ash, %	7.68	13.90	14.25	13.77
Ca, %	1.85	4.32	4.46	4.35
P, %	0.52	0.49	0.47	0.42
Xanthophylls, mg/kg	6.07	32.08	29.10	29.31
ME, kcal/kg	2760	2764	2800	2800
Vit. D_3_, U.I	2345	2344	2342	2342
Vit. HY D, ng/kg	25.13	25.12	25.10	25.10

### Laying hen performance

Productive performance data were collected on a replicate basis. Body weight (BW) was measured individually when the hens were 25, 34, 50, and 66 weeks of age (Table S1). Egg production (EP) and mortality (M, %; registered from 25 to 66 weeks of age) were recorded daily. Feed intake (FI) were measured on a replicate basis every fourteen days from 25 to 66 weeks of age. EP was calculated by dividing the number of daily eggs by the number of hens on the same day. Egg mass (EM) was calculated by multiplying egg weight by EP. The magnitude of production variables such as egg production were adjusted for hen mortalities.

Ca intake (IN, g d^–1^) was assessed considering the chemical composition of the diet and the feed intake of the different groups. Ca Output was estimated measuring the Ca of the eggshells (OUT, g d^–1^) in the different groups estimated by the percentage of calcium in the shell (~ 34%)^49^ of a hen egg and from the weight of the shell recorded in the qualitative analysis of the egg; finally, Ca OUT/IN has been estimated as a figure for Ca imbalance: higher values indicates a higher Ca imbalance. The possible Ca intake through soil ingestion (and little stones in the outdoor runs) was also hypothesized and Ca OUT/IN adjusted for the expected voluntary intake was added (9% of total consumption with a mean value of about 14% CaCO_3_H)^45^.

### Sampling

**Figure 5:**
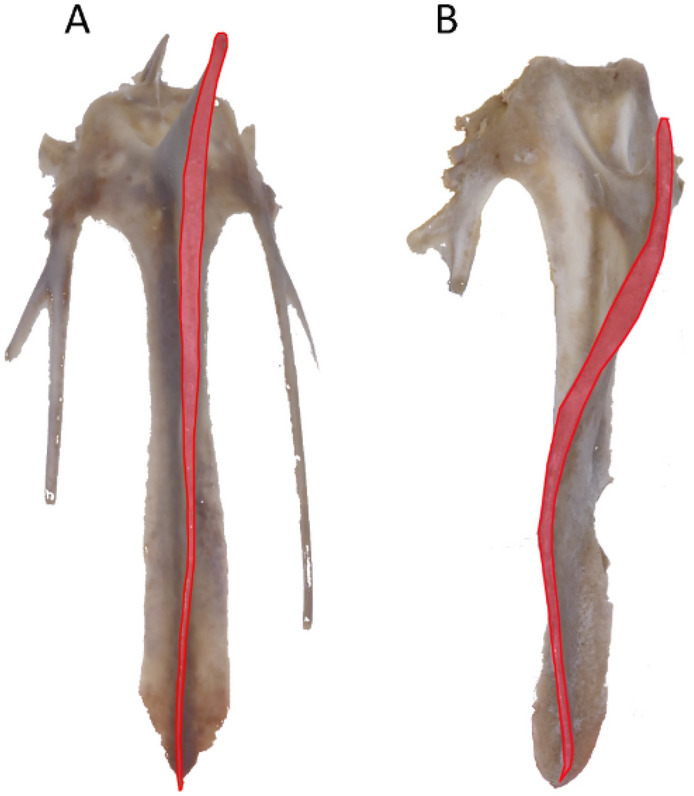
(**A**) Normal and (**B**) curved shape of *carina sterni* (in red) in keel bone of laying hens.

The structure selected for shape analysis was the *carina sterni* of the keel bone (Fig. 5). At 66 weeks of age fifteen individuals per group were selected and slaughtered (n = 75) in a commercial slaughterhouse 12 h after feed withdrawal. The animals were electrically stunned (110 V; 350 Hz) before being killed. After bleeding, the carcasses were plucked and eviscerated (nonedible viscera, including intestines, proventriculus, gall bladder, spleen, esophagus, and full crop were removed), and the *carina sterni* were removed.

Fifty-seven undamaged keel bones were transported to the Department of Agricultural, Environmental and Food Science of Perugia University laboratory, where they were boiled for 2 h in hot water to remove the meat and cartilages, then they were accurately washed to obtain the clean bone for photo acquisition. Eighteen bone samples were discarded as deformations have occurred during bone processing (e.g. cleaning and boiling).

### Geometric morphometrics analysis

**Figure 6:**
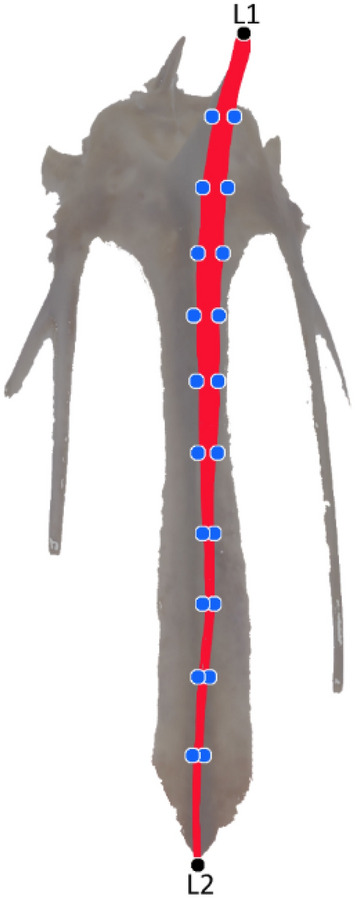
Landmarks (black circles, L1 and L2) and semi-landmarks (blue circles, N = 20) collected along the *carina sterni* of keel bone (in red).

Each bone was photographed in frontal view by a high-resolution camera (12 MP) set on a tripod. For each keel bone, landmarks (N = 2) and semi-landmarks (N = 20) were digitalized with TpsDig2.0 (Fig. 6). Landmarks were digitalized on the upper (L1) and lower (L2) edge of the keel bone. As it wasn’t possible to identify other homologous landmarks along the bone, to ensure shape coverage, 10 equally spaced semi-landmarks were automatically digitalized along each of the two outline curves recorded on the bone margin (from L1 to L2 and from L2 to L1).

Semi-landmarks could slide iteratively along the outline curves using the spline relaxation procedure algorithm of Bookstein^50,51^. After relaxation, semi-landmarks can be treated in multivariate analyses as homologous points. Landmarks and sliding landmarks were converted into shape coordinates using Procrustes superimposition^52^, removing information about location and orientation from the raw coordinates and standardizing each specimen to the CS. Residuals were analysed using the thin-plate spline (TPS) interpolating function^53^ producing principal warps.

Geometric Morphometrics analyses were performed using MorphoJ v.1.05^54^ freely available at https://morphometrics.uk/MorphoJ_page.html.

Centroid Size (CS), a measure of size in geometric morphometrics that is uncorrelated with shape for small isotropic landmark variation, was chosen as a proxy for keel bone length.

### Statistical analysis

Inter-group differences in hen final weight, keel-bone centroid size (CS), egg deposition rates and Ca traits (Ca intake, Ca output and their ratio, OUT/IN) were tested by means of Welch F test, in case of not-homogenous variances, and one-way ANOVA (with Tukey’s pairwise comparisons). A Hierarchical Procrustes ANOVA was carried out to test for the effect of genotype, rearing system, and of their interaction on size (CS) and shape (Procrustes Coordinates).

Categorical variables (KBD, KBF) were analysed with χ^2^.

To display shape variation of keel-bone, a principal component analysis (PCA), a simple statistical multivariate approach to summarize shape variables, was performed on the variance–covariance matrix of shape coordinates obtained by General Procrustes Superimposition^51^. Individuals were coloured in the plot according to (1) genotype and (2) productivity (i.e. classified as “high”, if > 90%, and “low”), to visualize shape differences according to these two categories of interest. Because precocity of egg deposition was collinear with the deposition rate it has not been considered.

As clean information on keel-bone shape differences among genotypes was not achieved through PCA, Canonical Variate Analysis (CVA), a supervised multivariate statistical analysis, was performed to better separate a priori known groups, providing an ordination that maximised intergroup separation respect to intragroup one.

A bivariate linear regression (method “robust”) was applied to describe the relationships between weight and shape and between size and shape. Scores along the first PCA axis, PC1, were chosen as a proxy for shape, CS data as a proxy for size. Weight data were log_10_ transformed.

Welch F test, one-way ANOVA, and linear regressions were performed using the freeware data analyser Past v. 4.03. Hierarchical Procrustes ANOVA, PCA, and CVA were performed using MorphoJ v.1.05.

To describe the patterns of covariation of keel-bone morphology in relation to the following grouping variables:

1. Number of fractures and deviations
2. Egg deposition rates and calcium balance

A partial least squares (PLS) analysis^55,56^ was performed. For details and computational aspects see Ref.^57,58^.

PLS analysis was performed using tpsPLS v 1.19^52^.

### Supplementary Information

Supplementary Information.

## Data Availability

The datasets used and/or analysed during the current study is available from the corresponding author on reasonable request.
