# Supplementary material for: Shape and fractures of carina sterni in chicken genotypes with different egg deposition rates reared indoor or free-range
Source: Sci Rep. 2023 Dec 15;13:22495. doi: 10.1038/s41598-023-49909-1 (PMC10728074; doi:10.1038/s41598-023-49909-1)
Supplement: Supplementary file 1 — Supplementary Information. [file 41598_2023_49909_MOESM1_ESM.docx]

**Supplemental material**

**Table S1.** Body weight (mean ± S.D.; n=30/genotype/rearing system) at different ages (25, 34, 50, and 66 weeks) of hen genotypes reared in enriched cages and free-range system.

|  | |  | **25 wks** | **34 wks** | **50 wks** | **66 wks** |  |
| --- | --- | --- | --- | --- | --- | --- | --- |
|  | |  |  |  |  |  |  |
| **Genotype^§^** | |  |  |  |  |  |  |
| LB | |  | 1.750^d^ ± 0.12 | 1.874^d^ ± 0.16 | 1.953^d^ ± 0.15 | 1.931^d^ ± 0.17 |  |
| BP | |  | 1.873^c^ ± 0.24 | 2.029^c^ ± 0.24 | 2.142^c^ ± 0.27 | 2.202^c^ ± 0.30 |  |
| RM | |  | 2.396^b^ ± 0.16 | 2.640^b^ ± 0.20 | 2.718^b^ ± 0.29 | 2.767^b^ ± 0.28 |  |
| BP x S | |  | 2.348^b^ ± 0.24 | 2.591^b^ ± 0.30 | 2.763^b^ ± 0.31 | 2.718^b^ ± 0.36 |  |
| RM x S | |  | 2.821^a^ ± 0.20 | 3.085^a^ ± 0.23 | 3.283^a^ ± 0.30 | 3.278^a^ ± 0.33 |  |
| **Rearing system^§^** | |  |  |  |  |  |  |
| Cage | |  | 2.239 ± 0.45 | 2.454 ± 0.54 | 2.564 ± 0.60 | 2.649^a^ ± 0.59 |  |
| Free-range | |  | 2.241 ± 0.43 | 2.437 ± 0.55 | 2.589 ± 0.49 | 2.517^b^ ± 0.50 |  |
| **Genotype** | **Rearing system^§^** |  |  |  |  |  |  |
| LB | Cage | | 1.736 ± 0.12 | 1.818^e^ ± 0.18 | 1.936^e^ ± 0.16 | 1.959^e^ ± 0.19 |  |
| LB | Free-range | | 1.763 ± 0.12 | 1.931^de^ ± 0.13 | 1.971^e^ ± 0.15 | 1.903^e^ ± 0.14 |  |
| BP | Cage | | 1.856 ± 0.22 | 2.024^d^ ± 0.24 | 2.078^de^ ± 0.28 | 2.270^d^ ± 0.30 |  |
| BP | Free-range | | 1.889 ± 0.27 | 2.034^d^ ± 0.25 | 2.211^d^ ± 0.25 | 2.136^de^ ± 0.29 |  |
| RM | Cage | | 2.368 ± 0.15 | 2.695^c^ ± 0.19 | 2.745^c^ ± 0.36 | 2.812^c^ ± 0.32 |  |
| RM | Free-range | | 2.424 ± 0.17 | 2.585^c^ ± 0.19 | 2.691^c^ ± 0.20 | 2.723^c^ ± 0.23 |  |
| BP x S | Cage | | 2.377 ± 0.24 | 2.542^c^ ± 0.28 | 2.655^c^ ± 0.29 | 2.727^c^ ± 0.37 |  |
| BP x S | Free-range | | 2.318 ± 0.24 | 2.640^c^ ± 0.32 | 2.867^c^ ± 0.31 | 2.710^c^ ± 0.36 |  |
| RM x S | Cage | | 2.843 ± 0.23 | 3.203^a^ ± 0.24 | 3.402^a^ ± 0.31 | 3.463^a^ ± 0.31 |  |
| RM x S | Free-range | | 2.800 ± 0.17 | 2.971^b^ ± 0.17 | 3.164^b^ ± 0.25 | 3.094^b^ ± 0.24 |  |
| SE |  | | 0.20 | 0.22 | 0.26 | 0.28 |  |
| *p*-value  Genotype  Rearing system  Genotype x Rearing system | | |  |  |  |  |  |
|  |  |  | < 0.001 | < 0.001 | < 0.001 | < 0.001 |  |
|  |  |  | 0.912 | 0.361 | 0.579 | < 0.001 |  |
|  |  |  | 0.431 | < 0.001 | < 0.001 | 0.010 |  |

^§^Within a column of each table section (i.e., the main factors and their interaction), means marked with distinct superscripts are significantly different (*p* < 0.05). LB, Lohmann Brown; BP, Bionda Piemontese; RM, Robusta Maculata; BP x S, Bionda Piemontese x Sasso; RM x S, Robusta Maculata

| **a)** | **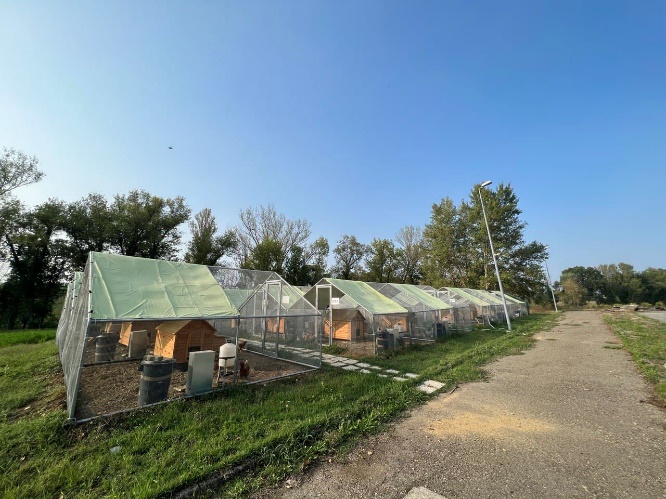** | **b)** | **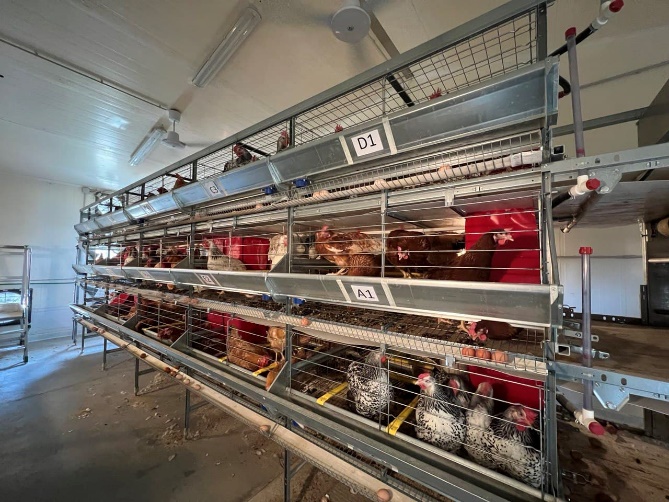** |
| --- | --- | --- | --- |
|  | **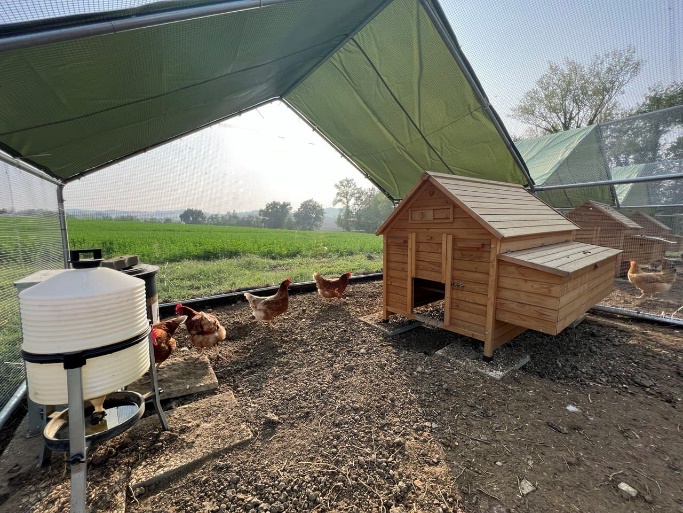** |  | **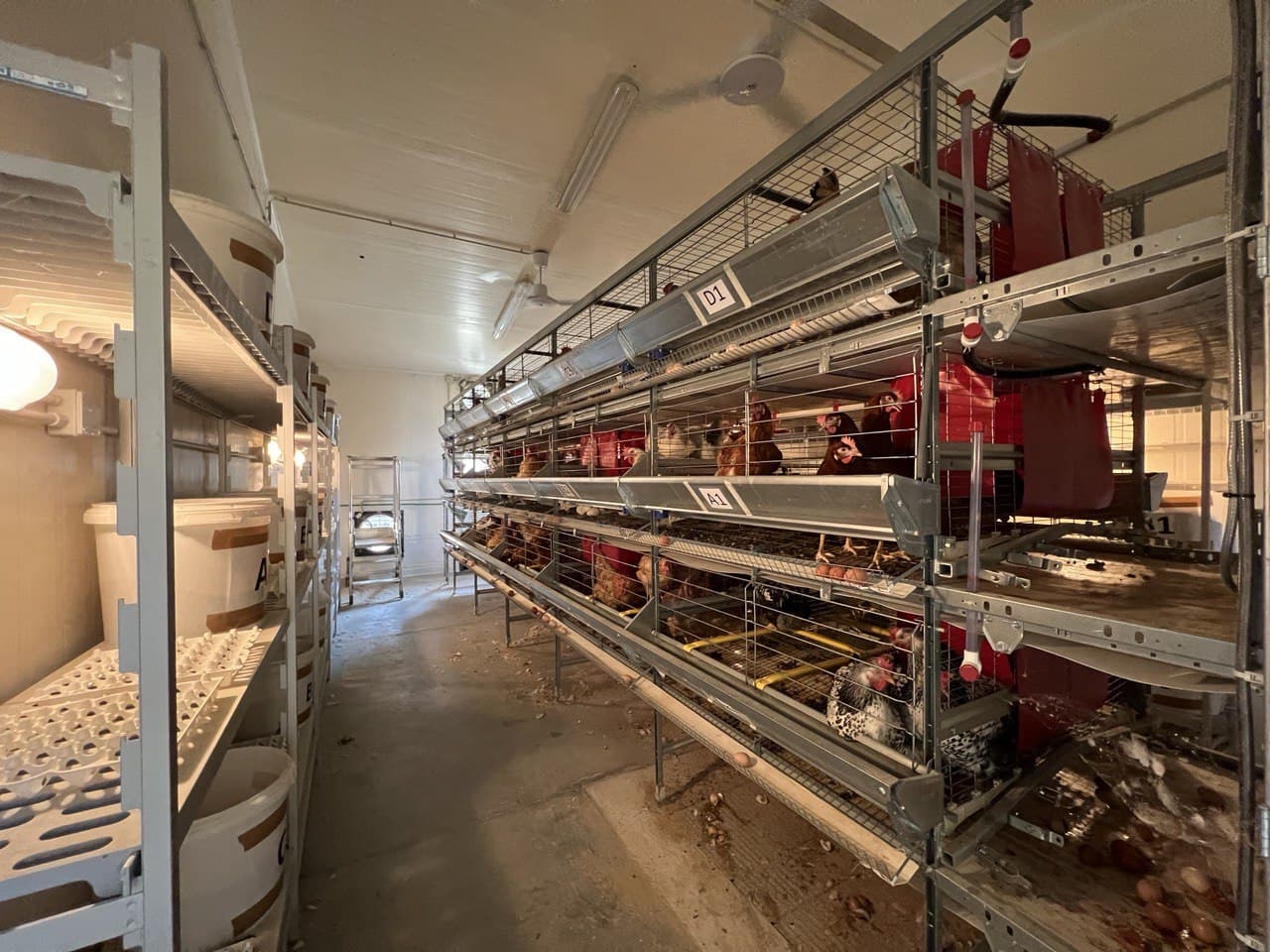** |

**Figure S1.** Pictures of two rearing systems analysed. A) free-range (FR) and b) enriched cages (EC)
